# Social learning of vocal structure in a nonhuman primate?

**DOI:** 10.1186/1471-2148-11-362

**Published:** 2011-12-16

**Authors:** Alban Lemasson, Karim Ouattara, Eric J Petit, Klaus Zuberbühler

**Affiliations:** 1 Ethologie Animale et Humaine, UMR 6552 - CNRS, Université de Rennes 1, Station Biologique, Paimpont, 35380, France; 2 Institut Universitaire de France, 103 bd Saint-Michel, Paris, 75005, France; 3 Laboratoire de zoologie et biologie animale, Université de Cocody-Abidjan, Abidjan 10, 10 BP770, Côte d'Ivoire; 4 Centre Suisse de Recherches Scientifiques, Taï Monkey Project, Abidjan 01, 01 BP1303, Côte d'Ivoire; 5 Ecobio, UMR6553 - CNRS, Université de Rennes 1, Station Biologique, Paimpont, 35380, France; 6 School of Psychology, University of St Andrews, St Andrews, KY16 9JP, UK

## Abstract

**Background:**

Non-human primate communication is thought to be fundamentally different from human speech, mainly due to vast differences in vocal control. The lack of these abilities in non-human primates is especially striking if compared to some marine mammals and bird species, which has generated somewhat of an evolutionary conundrum. What are the biological roots and underlying evolutionary pressures of the human ability to voluntarily control sound production and learn the vocal utterances of others? One hypothesis is that this capacity has evolved gradually in humans from an ancestral stage that resembled the vocal behavior of modern primates. Support for this has come from studies that have documented limited vocal flexibility and convergence in different primate species, typically in calls used during social interactions. The mechanisms underlying these patterns, however, are currently unknown. Specifically, it has been difficult to rule out explanations based on genetic relatedness, suggesting that such vocal flexibility may not be the result of social learning.

**Results:**

To address this point, we compared the degree of acoustic similarity of contact calls in free-ranging Campbell's monkeys as a function of their social bonds and genetic relatedness. We calculated three different indices to compare the similarities between the calls' frequency contours, the duration of grooming interactions and the microsatellite-based genetic relatedness between partners. We found a significantly positive relation between bond strength and acoustic similarity that was independent of genetic relatedness.

**Conclusion:**

Genetic factors determine the general species-specific call repertoire of a primate species, while social factors can influence the fine structure of some the call types. The finding is in line with the more general hypothesis that human speech has evolved gradually from earlier primate-like vocal communication.

## Background

What are the biological roots of vocal production learning in humans, a key capacity for the development of spoken language? Paradoxically, some cetaceans, songbirds and bats are more similar to humans in their vocal learning skills than any of the nonhuman primates, in the sense that all require social models to acquire functionally adequate vocal behavior [1]. The differences are not so much in terms of call use and perception, as there is good evidence that juvenile non-human primates learn from adult models how to use vocalizations in contextually appropriate ways [2-4]. In terms of call morphology, however, the default assumption has always been that nonhuman primate calls develop under strong genetic influences [5] with little or no voluntarily control [6]. Under this hypothesis, any kind of individual variability in call structure is usually explained in terms of maturational changes in morphology or differences in individuals' affective states. This hypothesis has also led to the widespread notion that studies of primate vocal behavior are largely irrelevant for understanding the evolution of speech.

Recently, however, this stance has become more controversial, largely due to a diverse body of evidence for vocal plasticity in non-human primates in the form of acoustic convergence at the group level (mouse lemurs [7], Japanese macaques [8], chimpanzees [9]) and individual level (marmosets [10], Campbell's monkeys [11]), as well as cases of apparent vocal innovation (Campbell's monkeys [12], chimpanzees [13]). These studies suggest that nonhuman primates must have some control over the acoustic structure of their calls and that some of their acoustic development can be socially guided. In humans, social affinity is a key factor responsible for vocal convergence [14-16].

Although the aforementioned primate studies are relevant, a common drawback in most of them is that they are typically unable to assess the impact of genetic and social factors separately, particularly social bonding. It is obvious that genetics will always determine the development of vocal production and processing systems, but what is less clear is how genetic factors interact with social ones in generating behavioural diversity. This problem has been hotly debated in many disciplines, particularly also in research on animal culture, and is thus not specific to this study [9,17,18]. Here, we were interested in the degree to which individual variation in primate calls could be explained with genetic and social factors. We tested the relative importance of these two factors on inter-individual acoustic variation in an arboreal, territorial, forest-dwelling primate, the Campbell's monkeys, which lives in one-male multi-female groups. Previous research has focused on the alarm calling behaviour of adult males [19,20], while the females' more quiet close range social calls have received relatively less attention [21]. Females maintain cohesion through the frequent emission of contact calls, which show individually distinct frequency contours. We were interested in the relative influence of social factors on these acoustic differences.

## Results

We addressed the issue with data collected during an 18-month field study with free-ranging Campbell's monkeys (*Cercopithecus campbelli campbelli*) in the Taï National Park, Ivory Coast. In many forest guenons, including Campbell's monkeys, females form the social core of the group and maintain individually differentiated long-term bonds with one another [22]. We focused on contact calls, which are exchanged between group members during friendly social interactions according to temporal and organizational rules [21,23]. Acoustically, contact calls vary individually, largely due to differences in the shape of the frequency modulation [11,21]. We sought to relate this acoustic variation to the genetic relatedness and their social affinities between individuals by comparing the adult females of two habituated groups. We then discuss these findings in relation to previous work on the same primate species by drawing comparisons to the social learning mechanisms observed in human vocal behavior.

**Figure 1:**
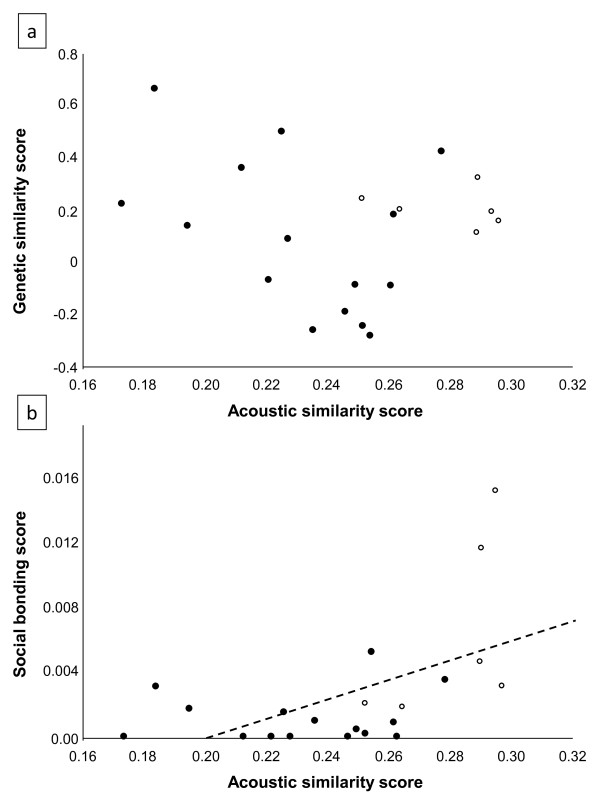
**Relationship between the acoustic similarities of female Campbell's monkeys' contact calls and their degree of social bonding regardless of genetic relatedness**. Black and white spots indicate dyads by members of the CAM1 and CAM2 group, respectively.

Our results showed that, if compared across dyads, the genetic similarities between females did not explain the acoustic similarities of calls (N = 21; Mantel test, r = -0.18, p = 0.882; Figure 1a). However, the acoustic similarities between two females' calls were significantly related to the amount of time they spent grooming each other, a widely accepted reliable indicator of bond strength in non-human primates [22] (Mantel test, r = 0.54, p = 0.001; Figure 1b).

## Discussion

Nonhuman primates may have little control over their basic call type repertoire, which appears to be largely species-specific and genetically determined. Within this repertoire, however, the different call types show various degrees of plasticity. This fact has been documented by a number of studies, although the underlying mechanisms responsible for this variation have remained poorly explored. One prediction from this research has been that calls with large frequency modulations and high importance in social interactions should be acoustically more flexible and as such less genetically-determined [21,24]. Our results support this hypothesis with a significant correlation between the strength of social bonds and the degree of acoustic similarity in a forest-dwelling nonhuman primate species, independent of their genetic relatedness. The key finding was that individuals with high degrees of social affinity also produced acoustically more similar calls.

We can think of at least three mutually exclusive hypotheses to explain this finding. Firstly, it is possible that individuals who are grooming each other experience the same affective state and thus produce similar sounds. This hypothesis predicts that the main acoustic differences are to be found during grooming and non-grooming sessions. Secondly, it is possible that individuals who produce acoustically similar call variants are simply more attracted to each other. Similarly, individuals who associate more often may have similar body sizes and, as a consequence, vocal tract morphologies and call structure. Thirdly, socially affiliated individuals may converge in the acoustic structure of calls they produced, a patterns seen in typical vocal learners, such as songbirds, bats, or cetaceans. In these species, vocal signatures may serve as "social badges", to advertise social bonds to each other and other group members [25]. Similar effects exist in spoken language, both during language development and in the form of vocal accommodation, a mechanism that serves in the maintenance of social bonds in adults [26].

When taking into account previous research on the same species, the first and second hypotheses are less likely compared to the third one. The first hypothesis can be reasonably excluded because all contact calls recorded in this study were uttered in non-grooming contexts. The second hypothesis is also unlikely because, in captivity, similarly sized individuals do not associate more often than others [22]. Moreover, adult females produce contact call variants with individualized frequency contours that change from one year to another, such that any given female can retain, loose or adopt call variants [11,27]. Other females discriminate between different call variants and respond as if they allocate them to specific callers [28]. Similar call variants can be produced by two or more females, especially individuals who play and interact peacefully with each other [11]. In captivity, such call variant sharing was found between individuals of very different ages (e.g. 3 vs. 14 years) or ranks (e.g. lowest vs. highest-ranking female), suggesting that morphological characteristics are poor predictors of differences in frequency contours [11]. It is also important to point out that intra-group agonistic interactions are relatively rare in this species, particularly between adult females [22]. Another relevant finding was that significant changes within a social group, such as the removal of a key individual, was associated with corresponding changes in the call patterns produced [11]. In sum, although body size affects a number of vocal characteristics in non-human primates [29], this variable alone cannot explain inter-individual differences in the frequency contours of call variants [24], the key acoustic variable in this study.

## Conclusions

Our study has shown that subtle acoustic variation in primate contact calls can be explained by social affiliation, but not by genetic relatedness. This is a relevant finding because it suggests that non-human primates can socially learn to alter the acoustic morphology of some of their calls. Although our findings are based on a relatively small sample size, their validity is high because they emerged from undisturbed, free-ranging individuals in their natural habitat. Future research will have to test this hypothesis with other primate species and, if possible, more individuals. We acknowledge that our conclusions are limited by the fact that similarities between genetic, acoustic and social variables were assessed with uni-variable indices (one set of genetic markers, fundamental frequency contours and grooming behavior). Although it is possible that other variables would have produced different findings, the ones chosen here are standard in behavioral studies when assessing genetic, vocal and social similarities. At this stage, results support the social learning hypothesis. Stronger evidence would require training one individual with a novel contact call variant to check whether this would spread to others, a manipulation commonly done in bird studies [25]. A more general point is that the links between genes and behavior are still poorly understood and generally restricted to cases of genetic malfunctioning (e.g. the FoxP2 "language" gene [30]) while the influence of normal allelic variation on behavior is not well understood. Although our findings do not suggest that acoustic similarities in Campbell's monkey contact calls are the result of genetic relatedness, the basic call repertoire is species-specific and as such genetically determined.

Our finding also has some comparative-evolutionary implications. In humans, the language 'melody' of parents' speech is known to influence the 'melody' of their newborn infant's cries [31]. In addition, dialectal characteristics of parental speech are acquired very early on, while friendships and social networks continue to influence the acoustic features of speech in later life [14,15]. Although the human speech signal is based on highly complex and elaborate acoustic maneuvers that goes significantly beyond changes in frequency contours, our data suggest that the evolutionary transition from vocal behavior to speech was the result of a gradual process along a common trajectory [32].

## Methods

### Study Groups

Data were collected in a study area adjacent to the research station of the Taï Monkey Project (5°50'N, 7°21'W), Ivory Coast, between January 2006 and September 2007 on two groups of Campbell's monkeys (CAM1, CAM2) that had been followed by researchers and field assistants for more than 10 years. Group members were fully habituated and individually known. Study groups consisted of one adult male each and six (CAM1) or four (CAM2) adult females with their offspring. The study was purely observational and non-invasive and ethically approved by the 'Office Ivoirien des Parcs et Reserves'.

### Observations and recordings

The observer (KO) carried out 15-min focal sampling on all adult females (mean +/- SE. = 16.00 +/- 0.58 hours of focal observations per female) of both groups in random order between 0800 and 1700 hours GMT. During observations, we recorded all contact calls (CH6 calls: [21]) emitted by a focal female. We also recorded the amount of time the focal animal was observed grooming or being groomed by another female. None of the calls recorded in this study were emitted during grooming interactions, but while females were travelling, foraging or resting. Recordings were made with a Sony TCDD100 DAT recorder and a Sennheiser ME88 microphone (sampling rate: 44.1 kHz; resolution: 16 bit). Fecal samples from all group members (2 or 3 exemplars per individual) were collected for subsequent genetic analyses.

### Genetic analysis

DNA was extracted from fecal samples and analyzed using human micro-satellites. Methods and genotyping results have been established and published elsewhere [33]. We used these data to calculate, for each pair of adult females, the Li's relatedness coefficient in SPAGeDi, a dyadic genetic similarity score [34,35]. The more positive the index, the more two individuals are genetically alike.

### Acoustic analysis

**Figure 2:**
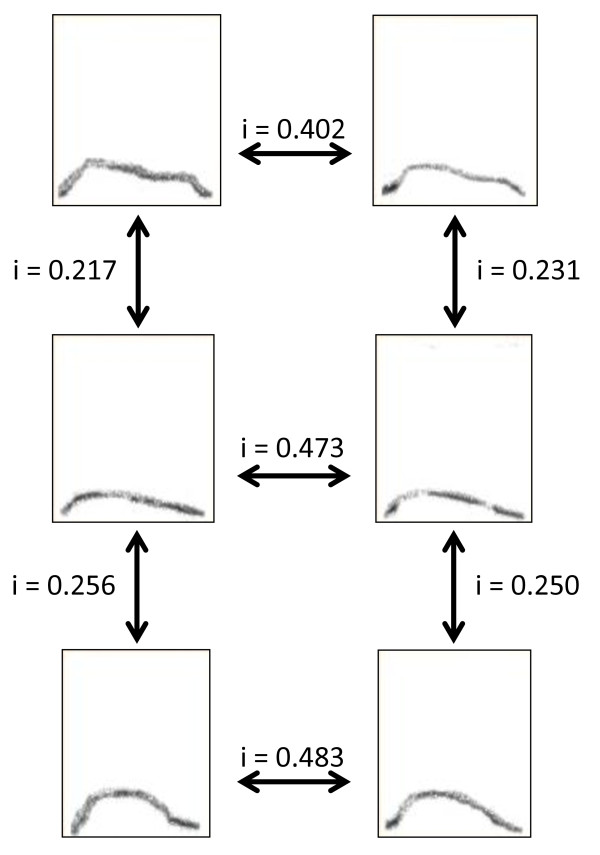
**Illustration of how similarity indices (i) were calculated between contact calls produced by different females**.

Using ANA Index acoustic software [36], we calculated acoustic similarity indices by comparing the shape of the arched frequency modulations of all CH6 calls (mean +/- SE = 19.0 +/- 3.3 calls per female). All comparisons were based on fundamental frequency patterns, not harmonics. A dyadic acoustic similarity score was obtained, for each pair of females within both groups, by averaging all the similarity indices obtained from the comparison of all of female A's calls with all of female B's calls (for details on the method and example of similarity index calculation see [11,27,28] and Figure 2). The similarity indices were based on pixel by pixel (size: 1 ms × 86 Hz) comparisons between pairs of spectrograms. Each pixel was associated with a grey value ranging from 0 to 255. If one or both compared pixels had a zero grey value, a score of 0 was given. If the two compared pixels differed by less than 16 in their grey values, a score of 2 was given. All other combinations were given a score of 1. The total of all scores was then divided by the total number of pixels in both spectrograms with a grey value above zero. This allowed us to generate a similarity index which ranged between 0 and 1. The algorithm then carried out the same operation for all possible superpositions by comparing spectrograms along the time axis, which generated similarity indices for each temporal position. Once all temporal positions had been compared, the algorithm determined the highest similarity index for the two spectrograms compared.

### Social bond analysis

We attributed a 'dyadic social bonding score' to each dyad by calculating the proportion of observation time two females were observed grooming each other. For example, the 'dyadic social bonding score' for the dyad AB was calculated by the total duration of all grooming events involving females A and B during focal observations divided by the total duration of all focal observations of females A and B.

### Statistical analysis

Because similarity indices were not independent of each other, we used Mantel tests to assess correlations between them [37]. We calculated three types of matrices for social, acoustic and genetic similarities between pairs of individuals. The same 21 pairs of females (N_CAM1 _= 15, N _CAM2 _= 6) were used in the three comparisons. Since both study groups contributed with a separate set of matrices, we combined them into a single one by replacing the diagonal empty cells (which corresponds to dyadic intergroup interactions that cannot occur) by barycenters [38]. The resulting three combined matrices were then compared with each other, using Mantel tests carried out with the R package [39].

## Authors' contributions

KO, AL and KZ conceived, designed and coordinated the study. KO collected the data. EP calculated the genetic indices. All authors interpreted and analyzed the data and drafted the manuscript. All authors read and approved the final manuscript.
